# Impact of surgeon experience on routine prolapse operations

**DOI:** 10.1007/s00192-017-3353-0

**Published:** 2017-06-02

**Authors:** Emil Nüssler, Jacob Kjær Eskildsen, Emil Karl Nüssler, Marie Bixo, Mats Löfgren

**Affiliations:** 1 Department of Clinical Science, Obstetrics and Gynecology, Umeå University, 90187 Umeå, Sweden; 2 Department of Management, School of Business and Social Sciences, Aarhus University, Aarhus, Denmark

**Keywords:** Pelvic organ prolapse, National register data, Patient-reported outcome, Surgical outcome, Quality control, Learning curve

## Abstract

**Introduction and hypothesis:**

Surgical work encompasses important aspects of personal and manual skills. In major surgery, there is a positive correlation between surgical experience and results. For pelvic organ prolapse (POP), this relationship has to our knowledge never been examined. In any clinical practice, there is always a certain proportion of inexperienced surgeons. In Sweden, most prolapse surgeons have little experience in performing prolapse operations, 74% conducting the procedure once a month or less. Simultaneously, surgery for POP globally has failure rates of 25–30%. In other words, for most surgeons, the operation is a low-frequency procedure, and outcomes are unsatisfactory. The aim of this study was to clarify the acceptability of having a high proportion of low-volume surgeons in the management of POP.

**Methods:**

A group of 14,676 exclusively primary anterior or posterior repair patients was assessed. Data were analyzed by logistic regression and as a group analysis.

**Results:**

Experienced surgeons had shorter operation times and hospital stays. Surgical experience did not affect surgical or patient-reported complication rates, organ damage, reoperation, rehospitalization, or patient satisfaction, nor did it improve patient-reported failure rates 1 year after surgery. Assistant experience, similarly, had no effect on the outcome of the operation.

**Conclusions:**

A management model for isolated anterior or posterior POP surgery that includes a high proportion of low-volume surgeons does not have a negative impact on the quality or outcome of anterior or posterior colporrhaphy. Consequently, the high recurrence rate was not due to insufficient experience of the surgeons performing the operation.

**Electronic supplementary material:**

The online version of this article (10.1007/s00192-017-3353-0) contains supplementary material, which is available to authorized users

## Introduction

Surgical work encompasses important aspects of both personal and manual skills, and it seems reasonable to assume that surgeons become better at a particular procedure by performing it more frequently. The discussion about the impact of operative volume on surgical results accelerated after Luft et al.’s article appeared in *The New England Journal of Medicine* in 1979 [1], which examined the impact of hospital surgical volume on patient mortality.

Recent research regarding the importance of the individual surgeon’s operative volume has generally been restricted to extensive, complicated procedures, and there seems to be a consensus that surgical experience is a critically important factor in cases of “major surgery” [2–4].

In obstetrics, a positive correlation between surgeon annual volume and decreased morbidity in Cesarean delivery was recently shown [5]. In gynecology, the association between operative volume and improved surgical outcome has been demonstrated regarding various types of hysterectomy [6–9]. This was confirmed in a recent meta-analysis examining hysterectomies, gynecological oncology, surgical mesh complications, and incontinence procedures [10]. In 2006, an assessment of a multitude of urogynecology procedures concluded that hospital and surgeon volumes possibly influence morbidity and mortality, but there were no conclusive results [11].

To date, an original article concerning solely the operation for pelvic organ prolapse (POP), which is one of the most common surgical interventions in gynecological practice, has not been published.

Women have an estimated lifetime risk of between 12% and 19% of undergoing surgery for POP [12–14] and the most common sites for repair are the anterior and posterior vaginal compartments.

The Swedish National Register of Gynecological Surgery (GynOp) has, since 2006, performed routine quality control of Swedish prolapse operations (www.gynop.org). During this monitoring process, it has become increasingly clear that a large percentage of surgeons in Sweden conduct POP operations at a very low rate (see Results). It seems reasonable to assume that organization of POP surgery in any international clinical setting includes a substantial number of surgeons with limited experience in routine prolapse surgery.

Simultaneously, failure rates are relatively high in POP surgery compared with other gynecological operations. Even the standard methods of repair, classic anterior and posterior colporrhaphy, generally have high recurrence rates, mostly within the range of 25–30% during the first year [15–17].

In other words, POP is a very common condition; most surgeons conduct the operation with a low frequency; and the operation has unsatisfactory outcomes.

The aim of this study was to clarify whether the operative results for isolated POP in the anterior or posterior compartment are associated with surgical experience, and, further, to clarify the acceptability of having a high proportion of low-volume surgeons in the management of POP.

The research questions were:

1. Does surgical experience influence complications, or the cure rate, of anterior and posterior colporrhaphy?
2. Does having an assistant surgeon with experience in the procedure improve the results of anterior and posterior colporrhaphy performed by an inexperienced surgeon?

## Materials and methods

This is a register-based study covering 9 years of consecutively registered POP operations. The data utilized were collected prospectively by GynOp from 1 January 2006 until 31 December 2015. The GynOp register includes all types of gynecological operations performed in Sweden and has, since 2006, registered POP operations on a national scale. Today, the register contains complete information on more than 45,000 prolapse procedures and the database is increasing by over 6,000 new cases a year. A comparison with the Swedish national patient register (where all Swedish surgical procedures are registered by law) shows that the GynOp coverage of prolapse operations is continuously above 95%.

The data collection process has previously been described [18, 19] and includes both surgeon- and patient-derived data up to 1 year after the operation. The data collection process involves both patients and operating surgeons. Preoperatively, patients complete a health declaration form and a validated questionnaire about prolapse symptoms [20]. Two months after the operation patients fill in a postoperative questionnaire about well-being and treatment-related complications. This questionnaire has previously been validated and provides complete post-treatment information [21]. Twelve months after the operation, patients fill in a final questionnaire about the results of the operation. The combination of a preoperative and two postoperative questionnaires makes it possible to analyze changes in patient-reported symptoms and outcomes. Our reported 12-month results are a combination of the 8-week and the 12-month questionnaire. We combine this information and control for double reporting. This sequential reporting gives us the possibility of distinguishing between short-term (<8 weeks) and long-term (8–52 weeks) complications. The gynecologist doing the preoperative assessment completes a form about preoperative, objective findings. In connection with the operation, the operating gynecologist records detailed information about the type and course of the operation and fills in a postoperative patient discharge form.

Furthermore, all patient questionnaires are reviewed by the operating gynecologist(s). All information from the patients, in addition to the surgeon’s evaluation, is recorded in the register.

We analyzed both patient-reported and surgeon-reported outcomes from the database. Patient-reported pain and complications were derived from the patient questionnaire 2 months after the operation. Patient satisfaction, functional parameters, and the feeling of protrusion were extracted from the 1-year questionnaire. Organ damage was reported by the surgeon either during the operation, at patient discharge, or in connection with the 2-month surgical evaluation. All medical complications, including re-operations, were registered by the surgeon, at the very latest in connection with the surgeon’s 1-year evaluation.

Very strict selection criteria were implemented to bolster the validity of the results. Only primary patients operated on for isolated prolapse, either in the anterior or the posterior vaginal wall, were included. Patients with all other types of POP, including prolapse in multiple compartments, recurrent prolapse, and prolapse in the apical compartment, were excluded.

Simultaneously, patients were only included if they had been in good health when they were operated on (category 1 or 2 patients, based on the American Society of Anesthesiologists [ASA] classification).

**Fig. 1:**
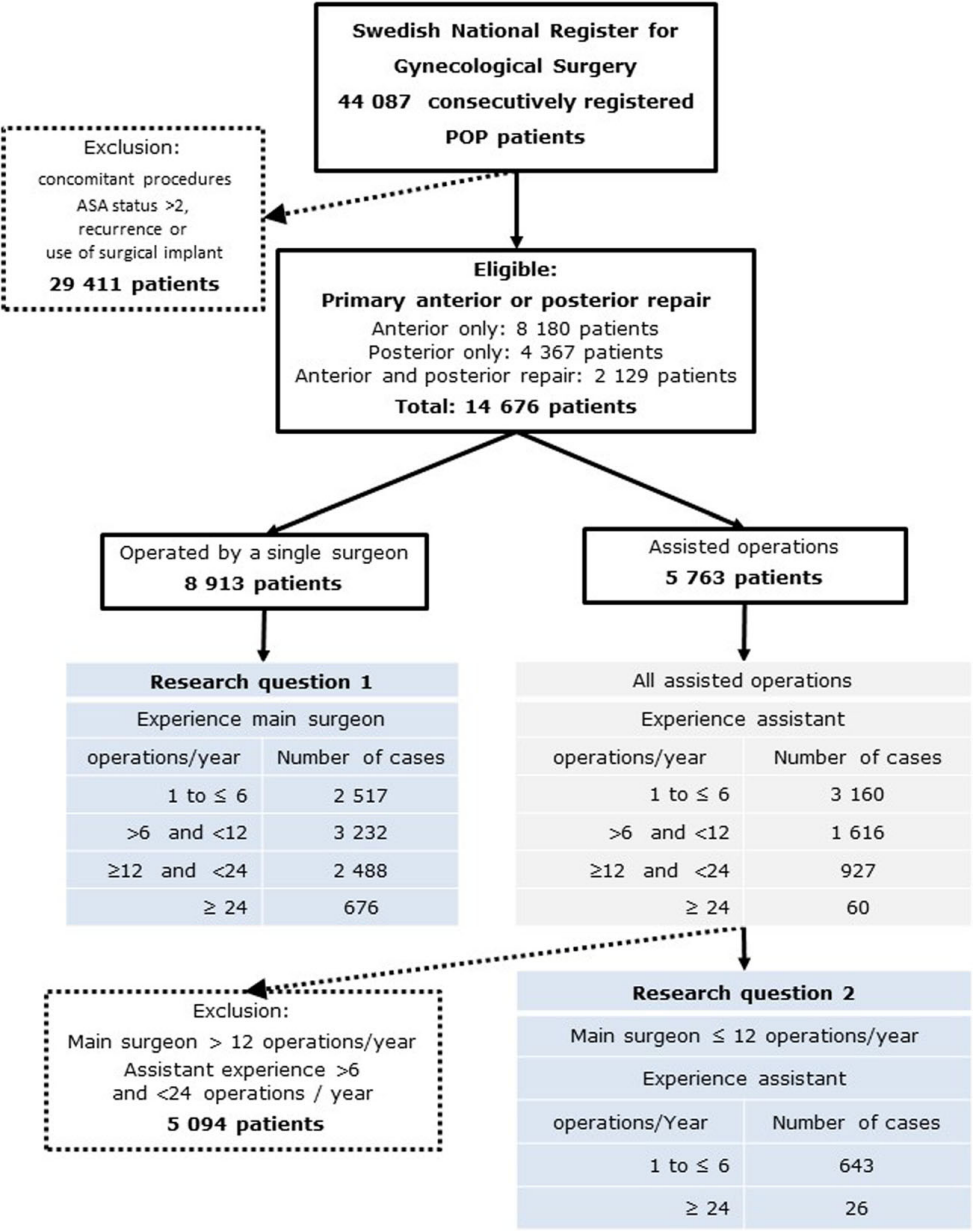
“ASA category” is a physical status classification system proposed and approved by the American Society of Anesthesiologists.* POP* pelvic organ prolapse

We excluded patients undergoing any other type of concurrent operations, regardless of type, including incontinence procedures and the use of surgical implants. This resulted in a uniform group of 14,676 healthy patients, operated on either by a single surgeon (8,913 patients) or by a surgeon/assistant team (5,763 patients), as shown in Fig. 1.

We analyzed the results of all surgeons who performed the operation at least once as the main surgeon during the observation period. An “active year” for a surgeon is defined as the calendar year in which she or he is registered at least once as the main surgeon. We defined “surgical volume” as the total number of all types of POP operations, in all active years, during which the surgeon acted either as the main surgeon or as the assistant surgeon. “Surgical experience” was subsequently calculated by dividing surgical volume by the number of active years, and shows the surgeon’s average number of operations as main surgeon or assistant per active year.

### Outcome measures

The only symptom specific to POP is the patient’s awareness of a vaginal bulge or protrusion. This is regarded as a valid way of measuring the presence of prolapse [22–25]. The cure rate, our main outcome measurement, therefore, was defined in terms of the absence or presence of a patient-reported feeling of a genital protrusion 1 year after surgery. In addition, we examined resource parameters (operation time and length of hospital stay), patient-reported parameters (number of days using painkillers at home; patient-reported complications within 8 weeks, requiring medical attention; rehospitalization; and patient-reported satisfaction 1 year after surgery), and surgeon-reported parameters: a composite of all surgeon-reported complications up to 8 weeks postoperatively; perioperative bleeding; organ damage; and rehospitalization within 1 year).

### Statistics

We analyzed the material in two steps, using logistic regression, and as a group analysis. Initial analysis applied logistic regression to examine the relationship between surgical experience and surgeon-reported reoperations or patient reported cure rate.

In the subsequent group analysis, data were handled in two different ways. When the dependent variable was interval- or quasi-interval-scaled, a univariate analysis of variance was performed. In those instances where the dependent variable was nominal-scaled, a Chi-squared test for independence was carried out. All analyses were carried out in SPSS 22 (SPSS, Chicago, IL, USA).

### Group analysis

For the performance analysis of research question 1, surgeons were divided into four groups according to their surgical experience, from between 1 and 6 (bottom group) to >24 operations (top group) per active year (Table 1), and subsequently compared. Results were extracted from unassisted operations to reflect individual surgeons’ results.

**Table 1:** Characteristics of patients undergoing single-surgeon operations

Surgical experience (umber of operations per active year)	≤ 6	>6 and < 12	≥12 and < 24	≥24	*P* value
Mean age, years (SD)					
Mean	63.53	62.04	62.26	64.41	
SD	10.93	12.10	11.54	11.04	0.000
*n*	2,517	3,232	2,488	676	
*N*	2,517	3,232	2,488	676	
BMI					
Mean	26.27	26.15	26.07	26.40	
SD	3.76	3.71	3.89	3.79	0.150
*n*	2,207	2,825	2,199	633	
*N*	2,517	3,232	2,488	676	
Parity					
Mean	2.54	2.58	2.52	2.50	
SD	1.09	1.16	1.12	1.084	0.158
*n*	2,250	2,867	2,220	646	
*N*	2,517	3,232	2488	676	
Position of the vaginal wall in relation to the hymen (cm)					
Mean	0.52	.71	0.82	−0.03	
SD	1.48	1.48	1.43	1.52	0.000
*n*	1,766	2,523	2,088	460	
*N*	2,517	3,232	2,488	676	
Smoking					
Yes	9.9%	9.9%	8.2%	7.9%	
95% CI	229	293	187	52	0.084
*n*	2,313	2,951	2,267	658	
*N*	2,517	3,232	2,488	676	
Preoperative estrogen					
Yes	46.1%	43.5%	44.4%	37.7%	
95% CI	(44.0–48.2)	(41.6–45.4)	(42.3–46.5)	(33.9–41.6)	0.002
*n*	2,214	2,798	2,190	631	
*N*	2,517	3,232	2,488	676	

Data are stratified by surgeons’ experience

*BMI* body mass index,* 95% CI* 95% confidence interval,* n* number of patients with information available,* N* total number of participants in each group,* SD* standard deviation

Analysis of research question 2, regarding the impact of an experienced assistant surgeon on operative results for surgeons with low experience, was done separately. This cohort consisted of all main surgeons with a maximum of 12 operations per active year, who were assisted by a fellow surgeon. Out of this cohort, we selected two groups for analysis based on the assistant surgeon’s experience: a low experience group where the assistant had little surgical experience, defined as a maximum of six operations per active year, and a high experience group where the assistant surgeon had ≥24 operations per active year (as in our definition of “experience”; Fig. 1). A performance analysis was conducted to compare the main surgeons’ results based on the assistant surgeons’ experience.

### Ethics

Ethics approval for the Swedish National Register for Gynecological Surgery (Dnr 04–107) and the present study, including its use of data from the register (Dnr 08-076 M), was granted by the Regional Ethical Review Board in Umeå, Sweden.

## Results

Data from the GynOp database show that, out of 1,092 surgeons who were active POP surgeons during 2006–2014, a total of 803 surgeons (73%) participated in POP operations once a month or less in their active years.

In the single-surgeon cohort, patient characteristics were statistically comparable for all parameters except for age, degree of prolapse, and preoperative estrogen use. The most experienced surgeons, in general, treated patients who were slightly older, had a lower degree of prolapse, and were less likely to have taken preoperative estrogen (Tables 1, 2).

**Table 2:** Patient characteristics in patients undergoing operations by surgeon/assistant teams

	Assistant performing ≤ 6 POP operations/year as the main surgeon	Assistant performing ≥ 24 POP operations/year as the main surgeon	*P* value
Mean age, years (SD)			
Mean	62.63	65.62	
SD	11.93	10.18	0.209
*n*	643	26	
*N*	643	26	
BMI			
Mean	26.25	27.08	
SD	4.06	5.05	0.325
*n*	570	25	
*N*	643	26	
Parity			
Mean	2.53	2.46	
SD	1.02	1.07	0.739
*n*	574	26	
*N*	643	26	
Position of the anterior vaginal wall in relation to the hymen (cm)			
Mean	0.94	1.71	
SD	1.51	1.86	0.062
*n*	539	14	
*N*	643	26	
Smoking			
Yes	10.2%	12.0%	
95% CI	(7.9–12.9)	(2.5–31.2)	0.746
*n*	589	25	
*N*	643	26	
Preoperative estrogen			
Yes	46.7%	30.8%	
95% CI	(42.6–50.9)	(14.3–51.8)	0.11
*n*	565	26	
*N*	643	26	

Patients were operated on by an inexperienced main surgeon (performing no more than 12 colporrhaphy operations per active year) and an assistant surgeon. All patients are stratified by the assistant surgeon’s experience as the main surgeon

*POP* pelvic organ prolapse

The surgeon/assistant cohorts were comparable on all parameters.

### Research question 1

Logistic regression showed no association between surgical experience and surgeon-reported complications for both research questions (*P* = 0.463 and *P* = 0.128 for research questions 1 and 2 respectively). Similarly, no association was found between surgical experience and patient reported cure rate 1 year after the operation (*P* = 0.195 and *P* = 0.128 for research questions 1 and 2 respectively).

Subsequent group analysis showed an impact of surgical experience on resource parameters. Both the duration of the procedure and the length of the hospital stay were substantially reduced with increased surgical experience (Table 3).

**Table 3:** Surgeon-reported outcomes of single-surgeon operations

Surgical experience (number of operations per active year)	≤ 6	>6 and < 12	≥12 and < 24	≥24	*P* value
Perioperative bleeding (ml)					
Mean	32.97	30.26	29.33	31.08	
SD	37.26	32.53	34.08	29.42	0.004
*n*	2,092	2,887	2172	580	
*N*	2,517	3,232	2,488	676	
Reoperation due to complications within 1 year					
Yes	0.8%	0.7%	0.7%	0.7%	
95% CI	(0.5–1.3)	(0.4–1.0)	(0.4–1.1)	(0.2–1.7)	0.926
*N*	2,214	2,748	2,243	615	
Surgeon-reported complication (of any kind) within 1 year					
Yes	16.0%	15.5%	14.3%	13.5%	
95% CI	402	502	357	91	0.219
*N*	2,214	2,748	2,243	615	
Organ damage (perforation: bladder, urethra, or intestine)					
Yes	0.1%	0.1%	0.1%	0.0%	
95% CI	(0.01–0.3)	(0.01–0.22)	(0.025–0.35)	N/A	0.752
*N*	2,517	3,232	2,488	676	
Operation time (min)					
Mean	42.31	41.33	37.18	29.01	
SD	18.231	17.970	16.945	13.426	0.000
*n*	2,078	2,908	2,159	576	
*N*	2,517	3,232	2,488	676	
Time in hospital (days)					
Mean	0.64	0.47	0.43	0.11	
SD	0.840	0.848	0.699	0.474	0.000
*n*	2,381	3,159	2,437	663	
*N*	2,517	3,232	2,488	676	

Surgeon-reported parameters showed that perioperative blood loss was reduced with increased surgical experience. There was no difference in surgeon-reported number of complications across the four surgical experience groups (Table 3) or with regard to reoperation rates (Table 3). No specific complication was more prevalent in any of the groups, regardless of surgical experience.

There were very few instances of organ damage, and those that did occur were evenly distributed among the experience groups (Table 3). Patient-reported days of using painkillers at home was associated with surgical experience, and was diminished with increasing experience. All other patient-reported parameters, including patient satisfaction, rehospitalization, and patient-reported complications were not affected by the frequency of performing the procedure (Table 4).

**Table 4:** Patient-reported outcomes of single-surgeon operations

Surgical experience (number of operations per active year)	≤ 6	>6 and < 12	≥12 and < 24	≥24	*P* value
Number of days using painkillers at home after the surgery					
Mean	4.65	4.55	4.61	3.52	
SD	7.13	6.13	5.95	6.06	0.004
*n*	1,639	2,301	1,761	490	
*N*	2,517	3,232	2,488	676	
Patient-reported complications within 8 weeks with medical attention sought					
Yes	20.0%	18.1%	18.4%	15.9%	
95% CI	(18.3–21.8)	(16.7–19.6)	(16.8–20.1)	(13.1–19.1)	0.104
*n*	2,099	2,788	2,175	603	
*N*	2,517	3,232	2,488	676	
Complications needing hospitalization up to 8 weeks after surgery					
Yes	4.2%	3.7%	2.9%	3.1%	
95% CI	(3.4–5.2)	(2.9–4.5)	(2.2–3.7)	(1.8–4.9)	0.142
*n*	1,915	2,561	2,025	553	
*N*	2,517	3,232	2488	676	
Satisfaction, 1 year after the surgery					
Yes	77.1%	74.8%	77%	73.3%	
95% CI	(75.0–79.1)	(72.9–76.6)	(75.0–79.0)	(69.4–77.6)	0.139
*n*	1,740	2,167	1,760	471	
*N*	2,214	2,748	2,243	615	
Patient-reported feeling of genital protrusion, 1 year after surgery					
Yes	74.0%	72.8%	73.2%	71.8%	
95% CI	(71.8–76)	(70.84–74.7)	(70.9–75.2)	(67.6–75.8)	0.765
*n*	1,743	2,124	1,721	479	
*N*	2,214	2,748	2243	615	

Satisfaction and failure rate results are taken from the 1-year questionnaire, covering operations from 1 January 2006 to 31 December 2015

Patient-reported cure rate after 1 year was not associated with surgical experience (Table 4).

### Research question 2

Assistant experience did not influence perioperative blood loss, surgeon-reported complications or reoperation rates (Table 5).

**Table 5:** Surgeon-reported and patient-reported outcomes of operations performed by surgeon/assistant teams

Surgical experience of main surgeon: ≤12 operations/year			
	Assistant performing ≤ 6 POP operations/year as main surgeon	Assistant performing ≥ 24 POP operations/year as main surgeon	*P* value
Surgeon-reported outcomes			
Perioperative bleeding (ml)			
Mean	30.32	35.23	
SD	33.45	14.92	0.494
*n*	576	22	
*N*	643	26	
Reoperation within 1 year			
Yes	1.4%	0.0%	
95% CI	(0.65–2.68)	N/A	0.544
*n*	643	26	
*N*	643	26	
Surgeon-reported complication (of any kind) within 1 year			
Yes	15.1%	11.5%	
95% CI	(12.4–18.1)	(2.4–30.2)	0.619
*n*	643	26	
*N*	643	26	
Organ damage (perforation: bladder, urethra, or intestine)			
Yes	0%	0%	
95% CI	N/A	N/A	
*n*	643	26	
*N*	643	26	
Patient-reported outcomes			
Number of days using painkillers at home after surgery			
Mean	4.75	6.44	
SD	6.87	10.79	0.344
*n*	455	16	
*N*	643	26	
Patient-reported complications within 8 weeks, with medical attention sought			
Yes	21.6%	31.8%	
95% CI	(18.3–25.2)	(13.9–54.9)	0.250
*n*	565	22	
*N*	643	26	
Complications needing hospitalization up to 8 weeks after surgery			
Yes	2.7%	5.0%	
95% CI	(1.5–4.5)	(0.1–24.9)	0.586
*n*	512	20	
*N*	643	26	
Satisfaction, 1 year after the surgery			
Yes	72.1%	60.0%	
95% CI	(67.7–76.1)	(63.1–80.9)	0.241
*n*	462	20	
*N*	643	26	
Patient-reported feeling of genital protrusion, 1 year after surgery			
Yes	70.7%	56.5%	
95% CI	(66.3–74.9)	(23.2–65.5)	0.146
*n*	458	23	
*N*	643	26	

Satisfaction and failure rates are taken from the 1-year questionnaire, covering operations from 1 January 2006 to 31 December 2015

All outcomes are for otherwise healthy patients operated on by an inexperienced main surgeon (performing no more than 12 colporrhaphy operations per active year) and an assistant surgeon. Results are stratified by the assistant surgeon’s experience as the main surgeon

Patient-reported complications, patient satisfaction, or cure rate were not affected by the assistants’ experience either (Table 5).

## Discussion

For isolated anterior or posterior vaginal wall surgery, increasing surgical experience appears to save hospital resources. However, individual surgeon volume seems to have no measurable effect on the cure rate of routine colporrhaphy operations among healthy, low-risk patients. A plausible explanation would be that the high recurrence rate is not due to insufficient surgical training or practice, but is inherent in the method. The dated Manchester–Fothergill technique uses the existing degraded, torn connective tissue, and has in principle not undergone any fundamental change over 100 years [26]. In the last decade, mesh, reinforcing native tissue, has improved the success rate, but not without drawbacks [18, 27]. Further research aiming to improve cure rates for anterior and posterior colporrhaphy should therefore focus on improvement of the surgical method.

We studied isolated anterior or posterior colporrhaphy in healthy patients, as they are the simplest to operate on, and therefore complicated cases in this group are rare. Making a comparison without complicated cases minimizes fluctuations in operation complexity, and makes it possible to compare across experience groups, and to evaluate whether surgical experience (or lack thereof) explains the high recurrence rate.

Postoperative complication rates in our study population were comparable across all experience groups. In our analysis, we included all types of surgeon-reported complications. As this opens up the possibility of overlooking a particular type of complication related to surgical experience, we stratified all reported complications. No specific complication was more prevalent in any of the groups, regardless of surgical experience. Also, there were so few instances of organ damage that a valid statistical analysis was not possible. These results are not surprising, as we investigated a relatively simple surgical procedure performed in otherwise generally healthy patients, where major complications were not expected to occur.

Our definition of experience could precipitate a subgroup of surgeons who play a more “observing” role than that of an active surgeon, performing only very few operations as the main surgeon, and assisting or observing most of the time. A group such as this, arguably, would be hard to define in relation to their actual experience of the operations. We have analyzed our material with regard to such a subgroup, and found no such cases, as most surgeons all operate primarily by themselves, or assist in an assistant/trainee situation.

Another possible bias is that experience could influence the threshold for reporting complications. Assuming that less experienced surgeons are more prone to reporting surgical complications than their more experienced colleagues, the uniformity of the reported complication rates confirms the conclusion that experience had no impact on our cohort. The most experienced surgeons, in general, treated patients who were less likely to have taken preoperative estrogen. Estrogen is supposed to have a beneficial effect on the tissue quality, and therefore enhances the chances of success of the operation. Even though the absolute difference between groups is only 8.5%, this could represent a slight bias toward no difference in this study.

Highly experienced surgeons also treated older patients. As the mean age difference is only around 2.5 years, this seems highly unlikely to have influenced the results of the study.

We found resource parameters to be dependent on the surgeons’ experience. The mean operation time was reduced by 31.4% (about 13 min) in favor of the more experienced surgeons, which, in proportional terms, seems considerable.

The hospital stay was significantly shorter for patients who were operated on by surgeons with more experience. This may reflect that patients recovered faster and were able to return home sooner, but it may also simply have been a consequence of different practices in the different experience groups. Less experienced surgeons are, presumably, more cautious, and the difference in duration of hospital stay may have been a product of surgeons’ caution, rather than of the patients’ health.

Differences in perioperative bleeding, although statistically detectable because of the large amount of data, can arguably be dismissed as clinically irrelevant, as the absolute divergence was around 3.5 ml.

The only symptom specific to prolapse is the awareness of a vaginal bulge or protrusion [28]. This is regarded as being a valid way of measuring the existence of prolapse [22–24], and is used in our material as the conclusive parameter to ascertain if the operation has been successful.

Patient-reported cure rates have the inherent problem of not having been objectively verified by a physician. De novo prolapse in a new compartment, therefore, would be reported as a failed operation, even though it may be unrelated to the surgical procedure. This would overestimate the total amount of failure, but it would not influence the differences between experience groups. “Objective verification” by a surgeon would also risk being biased, particularly as in most cases, it would be the operating surgeon carrying out an evaluation of his/her own work.

Our study procedure did not include any randomization, and possible confounding factors must be considered. In this study, we analyzed the difference between surgeon groups of similar experience, thus reducing the variation due to individuals. Our patient groups were comparable concerning health status, body mass index (BMI), parity, and smoking, which are established risk factors associated with POP [28–30].

Degree of prolapse, which affects the complexity of the operation, is possibly a major confounding factor in our study. The most experienced surgeon group (surprisingly) operated on less advanced prolapses. We measured the position of the anterior or posterior vaginal wall in relation to the hymen, in centimeters for each group. Although statistically significant, the largest absolute difference between groups was around 8 mm. Consequently, it seems highly unlikely that such a minimal discrepancy would affect the complexity of the operation or have clinical consequences.

Patients reported less postoperative pain with increasing surgeon experience. We used “patient-reported days of using painkillers” as a quantitative measurement. This method has the disadvantage of not necessarily being correlated with actual pain, as the use of painkillers may be the result of the instructions regarding pain management that the patient has received from the clinic.

It seems logical to assume that an inexperienced surgeon’s results will be improved if she or he is supervised by a highly experienced colleague. Our results contradict this assumption, but are consistent with the finding that the results of surgeons operating alone did not improve with surgical experience.

## Conclusions

A management model for low-risk, isolated anterior or posterior POP surgery that includes a high proportion of low-volume surgeons does not have a negative impact on the quality or outcome of anterior or posterior colporrhaphy.

Surgical experience had an effect on resource parameters, but did not influence the complication or cure rate. The presence of an experienced assistant surgeon did not improve the results of operations performed by surgeons with less experience. Therefore, it appears that the high recurrence rate was not due to insufficient experience of the surgeons performing the operation. Further research should focus on systematic and fundamental improvement of the surgical method.

## Electronic supplementary material

ESM 1 (JPEG 45 kb)
