# Correction to: Impact of surgeon experience on routine prolapse operations

**DOI:** 10.1007/s00192-017-3525-y

**Published:** 2017-12-13

**Authors:** Emil Nüssler, Jacob Kjær Eskildsen, Emil Karl Nüssler, Marie Bixo, Mats Löfgren

**Affiliations:** 1 Department of Clinical Science, Obstetrics and Gynecology, Umeå University, 90187 Umeå, Sweden; 2 Department of Management, School of Business and Social Sciences, Aarhus University, Aarhus, Denmark

**Correction to: Int Urogynecol J**

10.1007/s00192-017-3353-0

The article "Impact of surgeon experience on routine prolapse operations", written by Emil Nüssler, Jacob Kjær Eskildsen, Emil Karl Nüssler, Marie Bixo, and Mats Löfgren, was originally published without open access. After online publication it was found that article should be an Open Choice and as an open access publication. Therefore, the copyright of the article has been changed to © The Author(s) 2017 and the article is forthwith distributed under the terms of the Creative Commons Attribution 4.0 International License (http://creativecommons.org/licenses/by/4.0/), which permits use, duplication, adaptation, distribution and reproduction in any medium or format, as long as you give appropriate credit to the original author(s) and the source, provide a link to the Creative Commons license, and indicate if changes were made.

The original article has been corrected.

