# Supplementary figures and images for: Impact of surgeon experience on routine prolapse operations
Source: Int Urogynecol J. 2017 Jun 2;29(2):297–306. doi: 10.1007/s00192-017-3353-0 (PMC5780527; doi:10.1007/s00192-017-3353-0)

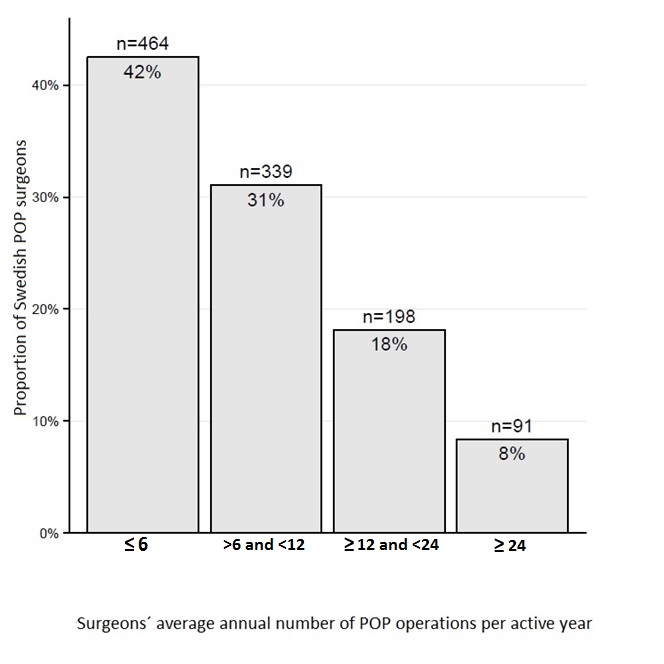

Supplement: Supplementary file 1 — (JPEG 45 kb) [file 192_2017_3353_Fig2_ESM.jpg]
